# Association of obesity with osteoporotic fracture risk in individuals with bone metabolism-related conditions: a cross sectional analysis

**DOI:** 10.3389/fnut.2024.1365587

**Published:** 2024-08-06

**Authors:** Guijun Yang, Hejun Jiang, Dan Xie, Shuhua Yuan, Jinhong Wu, Jing Zhang, Lei Zhang, Jiajun Yuan, Jilei Lin, Jiande Chen, Yong Yin

**Affiliations:** ^1^Department of Respiratory Medicine, Shanghai Children’s Medical Center, Shanghai Jiao Tong University School of Medicine, Shanghai, China; ^2^Department of Respiratory Medicine, Sanya Women and Children’s Hospital Affiliated to Hainan Medical College, Hainan Branch of Shanghai Children’s Medical Center, Shanghai Jiao Tong University School of Medicine, Sanya, Hainan, China; ^3^Medical Department of Shanghai Children’s Medical Center, Shanghai Jiao Tong University School of Medicine, Shanghai, China; ^4^Pediatric AI Clinical Application and Research Center, Shanghai Children’s Medical Center, Shanghai, China; ^5^Shanghai Engineering Research Center of Intelligence Pediatrics (SERCIP), Shanghai, China; ^6^Child Health Advocacy Institute, China Hospital Development Institute, Shanghai Jiao Tong University, Shanghai, China; ^7^Department of Respiratory Medicine, Linyi Maternal and Child Healthcare Hospital, Linyi Branch of Shanghai Children’s Medical Center, Shanghai Jiao Tong University School of Medicine, Linyi, Shandong, China; ^8^Shanghai Children’s Medical Center Pediatric Medical Complex (Pudong), Shanghai, China

**Keywords:** obesity, osteoporotic fractures, bone metabolism, BMI (body mass index), WC (waist circumference)

## Abstract

**Introduction:**

This study aimed to investigate the individual and composite associations of different indices of obesity on osteoporotic fractures at three different sites among individuals affected by conditions influencing bone metabolism.

**Methods:**

Participants were included from the National Health and Nutrition Examination Survey (NHANES), a national cross-sectional survey. BMI and WC were used separately and in combination to evaluate the presence of obesity. Obesity was defined as BMI ≥ 30 kg/m^2^, WC ≥ 88 cm in females, and WC ≥ 102 cm in males. Associations between obesity and osteoporotic fractures were assessed using multivariable logistic regression and OR curves. Associations modified by age, sex, race, and alcohol consumption were also evaluated.

**Results:**

A total of 5377 participants were included in this study. In multivariable logistic regression analyses, we found that BMI, WC, BMI defining obesity, and WC defining obesity were negatively associated with hip fracture (all *p* < 0.05). However, harmful associations between WC and BMI defining obesity and spine fracture were found (all *p* < 0.05). OR curves revealed that BMI and WC had a linear relationship with hip and spine fractures (all P for non-linearity >0.05). Further analyses showed that the highest WC quartile was harmfully associated with a higher risk of spine fractures (*p* < 0.05). Obese participants diagnosed by both BMI and WC were less likely to have hip fractures but more likely to have spine fractures (all P for trend <0.05). A significant interaction between age (Ref: age < 50 years) and BMI and WC was detected for hip fractures (all P for interaction <0.05).

**Discussion:**

In people with conditions influencing bone metabolism, obesity diagnosed by BMI and WC was associated with a lower risk of hip fracture, while obesity diagnosed by BMI and the highest WC quartile were associated with a higher risk of spine fracture.

## 1 Introduction

Osteoporotic fractures present a significant health and economic burden globally. Research indicated that these fractures contribute to 0.83% of the overall burden of noncommunicable diseases. About one-third of women over 50 years and one-fifth of elderly men will experience osteoporotic fractures in their lifetime (1, 2). Following osteoporotic fractures, excess mortality rates reach 9% in women and 24% in men after 1 year, escalating to 24% in women and 26% in men after 5 years (3). The wrist, hip, and spine represent the most prevalent sites of osteoporotic fractures (4). Conditions such as cancer, thyroid diseases, rheumatoid arthritis, and liver diseases significantly impact bone metabolism (5), leading individuals affected by these conditions to have poorer bone health compared to the general population. Consequently, they exhibit a higher propensity for osteoporotic fractures, with a more challenging healing process (6).

Anomalous bone metabolism has been associated with obesity (7, 8). Nearly every organ system is affected by obesity, encompassing cardiovascular, metabolic, pulmonary, gastrointestinal, and skeletal systems (9). However, the effects of obesity on osteoporotic fractures remain contentious. Traditionally, obesity has been suggested as protective against osteoporotic fractures. This effect may be explained by a mechanical load followed by a higher bone mineral density and a cushioning effect around the hip due to increased aromatase activity, influencing free sex hormones (10–13). A study indicated that no significant connections were found between obesity and the risk of major osteoporotic fractures (14). Some studies even suggested that obesity was linked to an increased risk of osteoporotic fractures (15–17). The potential mechanism behind this could be the excessive release of a series of cytokines (IL-6, TNF-α) leading to a negative impact on bone due to obesity’s proinflammatory state (8, 18).

Body mass index (BMI) and waist circumference (WC) serve as common surrogate measures of adiposity in clinical and public health practice (19). BMI and WC demonstrated varying relationships with osteoporotic fractures in some of the previously mentioned studies (13, 14). Research such as the study by Tao et al. found that weight-adjusted waist index (WWI) significantly correlates with an increased prevalence of hip and spine fractures, indicating the importance of differentiating fracture risk assessments based on specific locations (20). Furthermore, a Mendelian randomization study by Du et al. demonstrated a positive causal relationship between waist-to-hip ratio (WHR) and bone mineral density (BMD), suggesting that central obesity may influence BMD and thereby affect fracture risks differently across skeletal sites (21). Additionally, Li et al. reported in a Changsha-based study that the relationship between BMI and osteoporotic fractures varies significantly with fracture location, highlighting the nuanced impact of obesity on bone health (22). Moreover, Kim et al. demonstrated that the relationship between BMI and hip fracture risk shows a U-shaped curve in women and a reverse J-shaped curve in men, indicating that both low and high BMI levels are associated with increased fracture risks (23). These findings underscore the need for a holistic view of body composition and fat distribution when assessing osteoporotic fracture risks. However, most of the aforementioned studies exclude individuals affected by diseases influencing bone metabolism and solely examine the individual effect of BMI and WC on osteoporotic fractures. Therefore, studying the individual and combined associations of BMI and WC with osteoporotic fractures in populations affected by bone metabolism-affecting diseases is still necessary.

Hence, our objective was to assess the associations between obesity (defined by BMI and WC) and the incidence of osteoporotic fractures at the hip, wrist, and spine within a population drawn from the National Health and Nutrition Examination Survey (NHANES) spanning from 2005 to 2010, 2013 to 2014, and 2017 to 2018. Additionally, a secondary objective was to analyze these associations concerning age, sex, race, and alcohol consumption.

## 2 Materials and methods

### 2.1 Study population

The National Center for Health Statistics Ethics Review Board approved the protocols for the National Health and Nutrition Examination Survey (NHANES), with written informed consent obtained from all participants. As per the National Institutes of Health policy, the analysis conducted using de-identified data, which did not involve direct contact with participants, was not considered a human subjects study and was therefore not subject to institutional review board review. The NHANES surveys are representative cross-sectional studies developed by the National Center for Health Statistics (NCHS). The database utilized a complex, multi-stage, stratified, clustered probability sampling method, aiming to select a representative sample of civilians, rather than employing a simple random sample, based on the US population.

**Figure 1:**
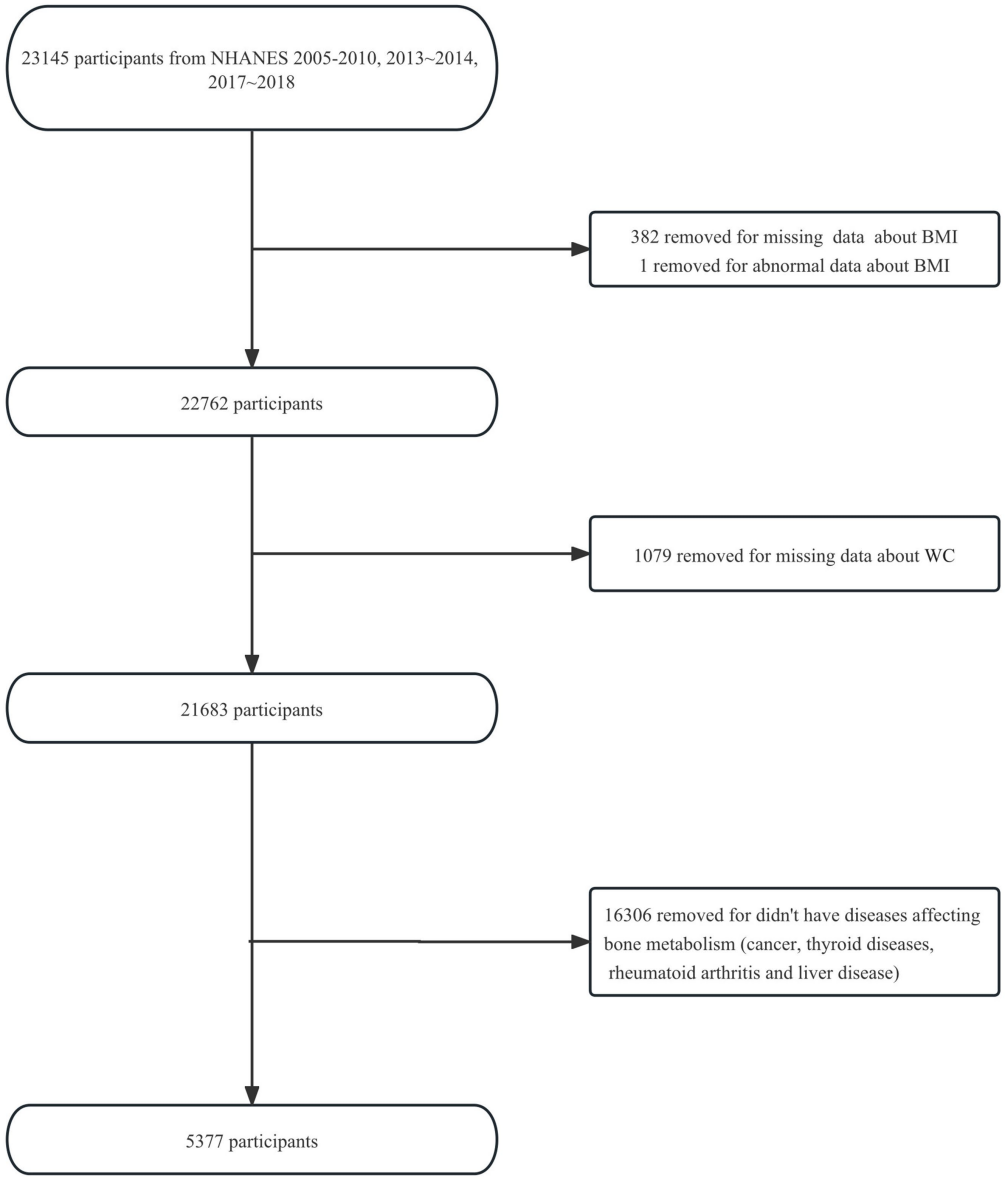
Flowchart of participants’ selection. BMI, Body Mass Index.

Five NHANES cycles (2005–2006, 2007–2008, 2009–2010, 2013–2014, 2017–2018) were selected for available information on osteoporotic fractures and bone mineral density (BMD) data. This selection was made because we want to further investigate whether BMD falls somewhere in the causal chain between obesity and osteoporotic fractures in the future. Initially, we included 23,145 participants aged ≥20 years (participants aged ≥85 years were recorded as 85 years) from the five NHANES cycles. After excluding 382 participants with missing data about BMI and 1 participant with an abnormal BMI of 130.21 kg/m^2^ (more than three times the standard deviation), 1,079 participants with missing data about WC, and 16,306 participants who did not have diseases affecting bone metabolism (cancer, thyroid diseases, rheumatoid arthritis, or liver disease), 5,377 eligible participants were enrolled in the study. The flowchart of the participant selection is shown in Figure 1.

### 2.2 Data extraction

The collected data included demographic information, underlying diseases, living habits, laboratory results, osteoporotic fractures, and physical measurements. Variables such as age, sex, race/ethnicity (Mexican American, Other Hispanic, non-Hispanic White, non-Hispanic Black, and other Race-Including Multiracial), educational level [Less than 9th grade, 9-11th grade (Includes 12th grade with no diploma), High school graduate/GED or equivalent, Some college or AA degree, College graduate or above, Not reported], poverty income ratio (PIR), drinking behavior (4/5 or more drinks every day, no 4/5 or more drinks every day, not reported), vitamin D, total protein, serum calcium, serum uric acid, cholesterol, serum phosphorus, and blood urea nitrogen were included as covariates. Detailed information about the covariates can be found on the NHANES website.

### 2.3 Osteoporotic fractures

The outcomes of this study were “osteoporotic fracture,” “hip fracture,” “wrist fracture,” and “spine fracture,” reported by a doctor, which were collected through personal interviews. The NHANES interviews were completed by trained interviewers in participants’ homes using the Computer-Assisted Personal Interview (CAPI) system.

“Hip fracture,” “wrist fracture,” and “spine fracture” were transformed into binary variables. Descriptions of the original values such as ‘Refused,’ ‘Do not know,’ or ‘Missing’ were converted to ‘No,’ while ‘Yes’ remained unchanged. If all the processed values for these three variables are ‘No,’ then the value of the variable ‘osteoporotic fracture’ is ‘No’; otherwise, it is ‘Yes’.

### 2.4 Obesity indices

BMI and WC were the primary obesity indices in this study. BMI was calculated as weight in kilograms divided by height in meters squared and then rounded to one decimal place. Weights and heights were collected at the Mobile Examination Center (MEC) by trained health technicians. The body weight data for participants who had limb amputations was set to “missing.” WC was measured as the circumference just above the right iliac crest at the midaxillary line. For women who were pregnant at the time of measurement, WC was recorded as “missing.”

BMI and WC were used as continuous variables. Participants (*N* = 5,377) were categorized into obese and not obese using BMI and WC. When BMI was used, both male and female participants were categorized according to the same criteria: obese (BMI ≥ 30 kg/m2), not obese (BMI < 30 kg/m2). When WC was used, male and female participants were categorized using different criteria: women were categorized as obese (WC ≥ 88 cm), not obese (WC < 88 cm); men were categorized as obese (WC ≥ 102 cm), not obese (WC < 102 cm) (24). Further categorizing obesity based on BMI and WC, BW-0 was defined as both BMI and WC diagnosed as not obese, BW-1 as BMI or WC diagnosed as obese, and BW-2 as both BMI and WC diagnosed as obese. WC was further stratified by quartiles in males and females separately: 1st quartile = WC-1 (< 95.0 cm in males; < 89.2 cm in females), 2nd quartile = WC-2 (95.0–104.8 cm in males; 89.2–99.2 cm in females), 3rd quartile = WC-3 (104.8–115.3 cm in males; 99.2–110.0 cm in females), and 4th quartile = WC-4 (≥ 115.3 cm in males; ≥ 110.0 cm in females).

### 2.5 Statistical analysis

All statistical analyses were performed using the statistical package R 4.2.2 (The R Foundation)^1^ and Free Statistics software versions 1.7.1. Continuous variables were presented as means ± standard deviation (SD). Categorical variables were analyzed by the χ^2^ test or the Fisher’s exact test, as appropriate. Multivariable logistic regression model was used to assess the relationship between obesity and osteoporotic fractures. The participants included in the study were divided into three groups based on the categories of obesity defined by both BMI and WC. An unadjusted model (model I) was first developed, followed by a fully adjusted model was calculated based on age, gender, race/ethnicity, educational level, poverty income ratio (PIR), drinking behavior, vitamin D, serum calcium, serum phosphorus, total protein, serum uric acid, cholesterol and blood urea nitrogen (model II). Analyses were then stratified by age, gender, race/ethnicity and drinking behavior. Then the interaction across subgroups was tested using the likelihood ratio test. OR curves were developed through restricted cubic spline models to examine the possible linear associations between BMI and WC and osteoporotic fractures, which was also conducted in total and age-specific participants. In these models, BMI and WC were used as continuous variable with five knots (5, 27.5, 50, 72.5 and 9th) suggested by Harrell (25). In the analysis, *p* values less than 0.05 were considered statistically significant.

## 3 Results

### 3.1 Study participants and baseline characteristics

This study included Americans aged ≥20 years, with an average age of 62.9 ± 13.8 years. 60.9% of the participants were male, and 39.1% were female. Among the 5,377 participants, 10.5% were Mexican American, 6.8% were other Hispanic, 59.6% were non-Hispanic white, 17.0% were non-Hispanic black, and 6.2% were other race (including multiracial). Participants were divided into three groups according to the obesity categories defined by both BMI and WC, as shown in Table 1. Participants’ characteristics differed significantly across confounders according to obesity categories. Non-Hispanic whites represented the predominant racial group in the study. However, within the various racial categories, Mexican Americans exhibited the highest prevalence of obesity, with 78% of this population being classified as obese. Among different educational levels, the highest prevalence of obesity was observed in the ‘Some college or AA degree’ category, with 75% of individuals in this group being classified as obese. The participants diagnosed as obese as defined by BMI and/or WC were more likely to be older, female, with lower vitamin D, total protein and serum calcium, and higher serum uric acid (Table 1).

**Table 1:** Characteristics of the study population based on obesity categories defined by both BMI and WC.

	Statistics	BW-0	BW-1	BW-2	p*
Number of subjects, *n* (%)	5,377	1,575 (29.3)	1,540 (28.6)	2,262 (42.1)	
Age (years), Mean ± SD	62.9 ± 13.8	62.4 ± 14.9	65.8 ± 13.3	61.2 ± 13.1	< 0.001
Gender, *n* (%)					< 0.001
Male	2,103 (39.1)	858 (54.5)	453 (29.4)	792 (35)	
Female	3,274 (60.9)	717 (45.5)	1,087 (70.6)	1,470 (65)	
Race, *n* (%)					< 0.001
Mexican American	564 (10.5)	124 (7.9)	156 (10.1)	284 (12.6)	
Other Hispanic	363 (6.8)	111 (7)	104 (6.8)	148 (6.5)	
Non-Hispanic White	3,203 (59.6)	940 (59.7)	989 (64.2)	1,274 (56.3)	
Non-Hispanic Black	916 (17.0)	243 (15.4)	206 (13.4)	467 (20.6)	
Other Race—Including Multiracial	331 (6.2)	157 (10)	85 (5.5)	89 (3.9)	
Education level, *n* (%)					< 0.001
Less than 9th grade	524 (9.7)	146 (9.3)	148 (9.6)	230 (10.2)	
9-11th grade (Includes 12th grade with no diploma)	739 (13.7)	211 (13.4)	204 (13.2)	324 (14.3)	
High school graduate/GED or equivalent	1,289 (24.0)	366 (23.2)	386 (25.1)	537 (23.7)	
Some college or AA degree	1,618 (30.1)	411 (26.1)	446 (29)	761 (33.6)	
College graduate or above	1,202 (22.4)	441 (28)	354 (23)	407 (18)	
Not reported	5 (0.1)	0 (0)	2 (0.1)	3 (0.1)	
4/5 or more drinks every day (%)					< 0.001
Yes	821 (15.3)	268 (17)	185 (12)	368 (16.3)	
No	3,595 (66.9)	1,038 (65.9)	1,050 (68.2)	1,507 (66.6)	
Not reported	961 (17.9)	269 (17.1)	305 (19.8)	387 (17.1)	
PIR, Mean ± SD	2.7 ± 1.6	2.8 ± 1.6	2.7 ± 1.6	2.5 ± 1.6	< 0.001
25-hydroxyvitamin D2 + D3 (nmol/L), Mean ± SD	72.1 ± 31.0	75.7 ± 30.6	75.8 ± 30.9	67.2 ± 30.7	< 0.001
Total protein(g/L), Mean ± SD	70.5 ± 4.9	70.8 ± 5.3	70.3 ± 4.8	70.4 ± 4.7	0.008
Serum total calcium (mmol/L), Mean ± SD	2.4 ± 0.1	2.4 ± 0.1	2.4 ± 0.1	2.3 ± 0.1	< 0.001
Serum uric acid(mmol/L), Mean ± SD	330.0 ± 87.3	309.2 ± 85.9	322.3 ± 84.2	349.5 ± 86.1	< 0.001
Cholesterol (mmol/L), Mean ± SD	5.0 ± 1.1	5.0 ± 1.1	5.1 ± 1.2	4.9 ± 1.1	< 0.001
Serum phosphorus (mmol/L), Mean ± SD	1.2 ± 0.2	1.2 ± 0.2	1.2 ± 0.2	1.2 ± 0.2	< 0.001
Blood urea nitrogen (mmol/L), Mean ± SD	5.5 ± 2.5	5.5 ± 2.5	5.6 ± 2.4	5.5 ± 2.6	0.539
BMI (kg/m^2), Mean ± SD	29.8 ± 7.0	23.3 ± 2.8	27.2 ± 2.0	36.2 ± 5.8	< 0.001
Obesity diagnosed by BMI, *n* (%)					< 0.001
No	3,093 (57.5)	1,575 (100)	1,518 (98.6)	0 (0)	
Yes	2,284 (42.5)	0 (0)	22 (1.4)	2,262 (100)	
WC (cm), Mean ± SD	102.6 ± 16.1	86.5 ± 8.7	99.3 ± 7.0	116.1 ± 12.6	< 0.001
Obesity diagnosed by WC, *n* (%)					< 0.001
No	1,597 (29.7)	1,575 (100)	22 (1.4)	0 (0)	
Yes	3,780 (70.3)	0 (0)	1,518 (98.6)	2,262 (100)	
Osteoporotic fracture, *n* (%)					0.128
No	4,502 (83.7)	1,302 (82.7)	1,313 (85.3)	1887 (83.4)	
Yes	875 (16.3)	273 (17.3)	227 (14.7)	375 (16.6)	
Hip fracture, *n* (%)					
No	5,253 (97.7)	1,521 (96.6)	1,507 (97.9)	2,225 (98.4)	**0.001**
Yes	124 (2.3)	54 (3.4)	33 (2.1)	37 (1.6)	
Wrist fracture, *n* (%)					
No	4,747 (88.3)	1,386 (88)	1,377 (89.4)	1984 (87.7)	0.253
Yes	630 (11.7)	189 (12)	163 (10.6)	278 (12.3)	
Spine fracture, *n* (%)					
No	5,167 (96.1)	1,518 (96.4)	1,491 (96.8)	2,158 (95.4)	0.068
Yes	210 (3.9)	57 (3.6)	49 (3.2)	104 (4.6)	

BW-0, BMI and WC diagnosed as not obese; BW-1, BMI or WC diagnosed as obese; BW-2, BMI and WC diagnosed as obese. PIR, poverty income ratio; BMI, body mass index; WC, waist circumference. *p-values were calculated using chi-square tests (for most categorical variables), Fisher’s exact test (for categorical variables with zero frequencies), and one-way analysis of variance (ANOVA) for continuous variables. The bold values indicate *p* < 0.05, signifying statistical significance.

### 3.2 Effects of obesity indices on osteoporotic fractures

The results of the multivariable logistic regression analysis are shown in Table 2. In the adjusted model II, BMI and obesity as defined by BMI were negatively associated with hip fracture (OR = 0.95 95%CI: 0.92 ~ 0.99, *p* = 0.006; OR = 0.6 95%CI: 0.39 ~ 0.93, *p* = 0.021). WC and obesity as defined by WC were also negatively associated with hip fracture (OR = 0.98 95%CI: 0.96 ~ 0.99, *p* = 0.002; OR = 0.45 95%CI: 0.3 ~ 0.67, *p* < 0.001). Age-specific stratified analyses revealed a protective association for BMI and WC and hip fracture within participants ≥50 years (OR = 0.94 95%CI: 0.91 ~ 0.98, *p* = 0.003; OR = 0.97 95%CI: 0.96 ~ 0.99, *p* = 0.001). There was sufficient evidence to suggest interaction between age (Ref: age < 50 years) and both BMI and WC for hip fracture (P for interaction = 0.039; P for interaction = 0.029; Table 3). Participants diagnosed as obese by both BMI and WC were less likely to have hip fracture (OR = 0.42 95%CI: 0.26 ~ 0.68, *p* < 0.001; P for trend <0.001) compared to those diagnosed as not obese by both BMI and WC. WC and obesity as defined by BMI were analyzed to have a harmful association with spine fracture (OR = 1.43 95%CI: 1.03 ~ 1.99, *p* = 0.034; OR = 1.01 95%CI: 1 ~ 1.02, *p* = 0.019). However, obesity defined by WC was found to have no correlation with spine fracture. (OR = 1.45 95%CI: 0.99 ~ 2.12, *p* = 0.057) Participants diagnosed as obese by both BMI and WC were found to be more likely to have spine fractures (OR = 1.59 95%CI: 1.06 ~ 2.4, *p* = 0.026; P for trend = 0.021) compared to those diagnosed as not obese by both BMI and WC. Meanwhile, the analysis of obesity indices in relation to osteoporotic and wrist fractures revealed no association (all *p* > 0.05).

**Table 2:** The association between obesity indices and osteoporotic fractures.

	F/A (%)	OR (95%CI) Model I	*p*	OR (95%CI) Model II	*p*
**Osteoporotic fracture**					
BMI	875/5377 (16.3)	1 (0.99 ~ 1.01)	0.497	1 (0.99 ~ 1.02)	0.532
**Obesity diagnosed by BMI**					
No	500/3093 (16.2)	1(Ref)		1(Ref)	
Yes	375/2284 (16.4)	1.02 (0.88 ~ 1.18)	0.804	1.11 (0.94 ~ 1.32)	0.212
WC	875/5377 (16.3)	1 (1 ~ 1.01)	0.483	1 (1 ~ 1.01)	0.247
**Obesity diagnosed by WC**					
No	273/1597 (17.1)	1(Ref)		1(Ref)	
Yes	602/3780 (15.9)	0.92 (0.79 ~ 1.07)	0.289	0.96 (0.8 ~ 1.16)	0.676
**BMI&WC-category**					
BW-0	273/1575 (17.3)	1(Ref)		1(Ref)	
BW-1	227/1540 (14.7)	0.82 (0.68 ~ 1)	0.049	0.84 (0.67 ~ 1.04)	0.108
BW-2	375/2262 (16.6)	0.95 (0.8 ~ 1.12)	0.539	1.03 (0.84 ~ 1.27)	0.743
Trend. test	875/5377 (16.3)	0.98 (0.9 ~ 1.07)	0.665	1.03 (0.93 ~ 1.14)	0.606
**Hip fracture**					
BMI	124/5377 (2.3)	0.95 (0.92 ~ 0.98)	0.001	0.95 (0.92 ~ 0.99)	**0.006**
**Obesity diagnosed by BMI**					
No	87/3093 (2.8)	1(Ref)		1(Ref)	
Yes	37/2284 (1.6)	0.57 (0.39 ~ 0.84)	0.004	0.6 (0.39 ~ 0.93)	**0.021**
WC	124/5377 (2.3)	0.98 (0.97 ~ 0.99)	0.001	0.98 (0.96 ~ 0.99)	**0.002**
**Obesity diagnosed by WC**					
No	54/1597 (3.4)	1(Ref)		1(Ref)	
Yes	70/3780 (1.9)	0.54 (0.38 ~ 0.77)	0.001	0.45 (0.3 ~ 0.67)	**<0.001**
**BMI&WC-category**					
BW-0	54/1575 (3.4)	1(Ref)		1(Ref)	
BW-1	33/1540 (2.1)	0.62 (0.4 ~ 0.96)	0.031	0.47 (0.29 ~ 0.77)	**0.002**
BW-2	37/2262 (1.6)	0.47 (0.31 ~ 0.72)	<0.001	0.42 (0.26 ~ 0.68)	**<0.001**
Trend. test	124/5377 (2.3)	0.68 (0.55 ~ 0.84)	<0.001	0.64 (0.49 ~ 0.82)	**<0.001**
**Wrist fracture**					
BMI	630/5377 (11.7)	1 (0.99 ~ 1.01)	0.967	1.01 (0.99 ~ 1.02)	0.325
**Obesity diagnosed by BMI**					
No	352/3093 (11.4)	1(Ref)		1(Ref)	
Yes	278/2284 (12.2)	1.08 (0.91 ~ 1.28)	0.373	1.17 (0.97 ~ 1.43)	0.103
WC	630/5377 (11.7)	1 (1 ~ 1.01)	0.251	1 (1 ~ 1.01)	0.203
**Obesity diagnosed by WC**					
No	189/1597 (11.8)	1(Ref)		1(Ref)	
Yes	441/3780 (11.7)	0.98 (0.82 ~ 1.18)	0.861	1.02 (0.83 ~ 1.26)	0.837
**BMI&WC-category**					
BW-0	189/1575 (12)	1(Ref)		1(Ref)	
BW-1	163/1540 (10.6)	0.87 (0.69 ~ 1.08)	0.212	0.87 (0.68 ~ 1.12)	0.294
BW-2	278/2262 (12.3)	1.03 (0.84 ~ 1.25)	0.787	1.11 (0.88 ~ 1.41)	0.36
Trend. test	630/5377 (11.7)	1.02 (0.93 ~ 1.13)	0.666	1.07 (0.95 ~ 1.2)	0.274
**Spine fracture**					
BMI	210/5377 (3.9)	1.01 (1 ~ 1.03)	0.139	1.02 (1 ~ 1.04)	0.116
**Obesity diagnosed by BMI**					
No	106/3093 (3.4)	1(Ref)		1(Ref)	
Yes	104/2284 (4.6)	1.34 (1.02 ~ 1.77)	0.036	1.43 (1.03 ~ 1.99)	**0.034**
WC	210/5377 (3.9)	1.01 (1 ~ 1.02)	0.024	1.01 (1 ~ 1.02)	**0.019**
**Obesity diagnosed by WC**					
No	57/1597 (3.6)	1(Ref)		1(Ref)	
Yes	153/3780 (4)	1.14 (0.84 ~ 1.55)	0.408	1.45 (0.99 ~ 2.12)	0.057
**BMI&WC-category**					
BW-0	57/1575 (3.6)	1(Ref)		1(Ref)	
BW-1	49/1540 (3.2)	0.88 (0.59 ~ 1.29)	0.501	1.19 (0.76 ~ 1.87)	0.447
BW-2	104/2262 (4.6)	1.28 (0.92 ~ 1.78)	0.138	1.59 (1.06 ~ 2.4)	**0.026**
Trend. test	210/5377 (3.9)	1.16 (0.98 ~ 1.37)	0.09	1.27 (1.04 ~ 1.56)	**0.021**

F/A, number of people with fracture (F) vs. number of all people (A). BW-0, BMI and WC diagnosed as not obese; BW-1, BMI or WC diagnosed as obese; BW-2, BMI and WC diagnosed as obese. BMI, body mass index; WC, waist circumference. Model I: Unadjusted model (univariate model). Model II: Adjusted for age, gender, race/ethnicity, educational level, poverty income ratio (PIR), drinking behavior, vitamin D, serum calcium, serum phosphorus, total protein, serum uric acid, cholesterol and blood urea nitrogen. The bold values indicate *p* < 0.05, signifying statistical significance.

**Table 3:** Subgroup analyses on the association between obesity indices (BMI and WC) and osteoporotic fractures (hip and spine).

	BMI				WC			
	F/A (%)	OR (95CI)	*p*	P-interaction	F/A (%)	OR (95CI)	*p*	P-interaction
**Hip fracture**								
**Subgroup analysis stratified by age**								
Age < 50	17/892 (1.9)	1.01 (0.93 ~ 1.09)	0.845	**0.039**	17/892 (1.9)	1 (0.97 ~ 1.04)	0.818	**0.029**
Age > = 50	107/4485 (2.4)	0.94 (0.91 ~ 0.98)	**0.003**		107/4485 (2.4)	0.97 (0.96 ~ 0.99)	**0.001**	
**Subgroup analysis stratified by gender**								
Male	41/2130 (1.9)	0.96 (0.9 ~ 1.03)	0.292	0.997	41/2103 (1.9)	0.99 (0.96 ~ 1.01)	0.298	0.599
Female	83/3274 (2.5)	0.95 (0.91 ~ 0.99)	**0.015**		83/3274 (2.5)	0.97 (0.96 ~ 0.99)	**0.003**	
**Subgroup analysis stratified by race**								
Mexican American	9/564 (1.6)	1.05 (0.94 ~ 1.16)	0.403	0.528	9/564 (1.6)	1 (0.95 ~ 1.05)	0.918	0.863
Other Hispanic	8/363 (2.2)	1.09 (0.93 ~ 1.28)	0.281		8/363 (2.2)	1.01 (0.93 ~ 1.09)	0.858	
Non-Hispanic White	86/3203 (2.7)	0.93 (0.89 ~ 0.97)	**0.002**		86/3203 (2.7)	0.97 (0.96 ~ 0.99)	**0.001**	
Non-Hispanic Black	12/916 (1.3)	1.04 (0.93 ~ 1.16)	0.522		12/916 (1.3)	1.03 (0.97 ~ 1.08)	0.327	
Other Race—Including Multiracial	9/331 (2.7)	0.87 (0.71 ~ 1.07)	0.182		9/331 (2.7)	0.94 (0.86 ~ 1.03)	0.2	
**Subgroup analysis stratified by 4/5 or more drinks every day (%)**								
Yes	26/821 (3.2)	0.96 (0.9 ~ 1.03)	0.279	0.611	26/821 (3.2)	0.99 (0.96 ~ 1.02)	0.445	0.686
No	75/3595 (2.1)	0.95 (0.91 ~ 0.99)	**0.025**		75/3595 (2.1)	0.97 (0.95 ~ 0.99)	**0.003**	
Not reported	23/961 (2.4)	0.95 (0.86 ~ 1.04)	0.274		23/961 (2.4)	0.98 (0.94 ~ 1.01)	0.217	
**Spine fracture**								
**Subgroup analysis stratified by age**								
Age < 50	31/892 (3.5)	0.98 (0.92 ~ 1.05)	0.613	0.306	31/892 (3.5)	1.01 (0.98 ~ 1.03)	0.72	0.555
Age > = 50	179/4485 (4)	1.02 (1 ~ 1.05)	0.09		179/4485 (4)	1.01 (1 ~ 1.02)	**0.023**	
**Subgroup analysis stratified by gender**								
Male	93/2103 (4.4)	1.03 (0.99 ~ 1.07)	0.119	0.751	93/2103 (4.4)	1.02 (1 ~ 1.03)	0.056	0.718
Female	117/3274 (3.6)	1.01 (0.98 ~ 1.04)	0.447		117/3274 (3.6)	1.01 (1 ~ 1.03)	0.122	
**Subgroup analysis stratified by race**								
Mexican American	15/564 (2.7)	1.04 (0.96 ~ 1.14)	0.321	0.878	15/564 (2.7)	1.02 (0.98 ~ 1.06)	0.396	0.557
Other Hispanic	11/363 (3)	0.94 (0.79 ~ 1.12)	0.484		11/363 (3)	0.98 (0.91 ~ 1.05)	0.502	
Non-Hispanic White	141/3203 (4.4)	1.01 (0.99 ~ 1.04)	0.348		141/3203 (4.4)	1.01 (1 ~ 1.02)	0.107	
Non-Hispanic Black	23/916 (2.5)	1 (0.94 ~ 1.07)	1		23/916 (2.5)	1.01 (0.98 ~ 1.04)	0.669	
Other Race - Including Multiracial	20/331 (6)	1.08 (0.98 ~ 1.19)	0.107		20/331 (6)	1.04 (1 ~ 1.08)	0.078	
**Subgroup analysis stratified by 4/5 or more drinks every day (%)**								
Yes	49/821 (6)	1.03 (0.98 ~ 1.09)	0.24	0.995	49/821 (6)	1.01 (0.99 ~ 1.04)	0.234	0.948
No	138/3595 (3.8)	1.01 (0.98 ~ 1.04)	0.351		138/3595 (3.8)	1.01 (1 ~ 1.03)	0.059	
Not reported	23/961 (2.4)	1.02 (0.95 ~ 1.11)	0.535		23/961 (2.4)	1.01 (0.97 ~ 1.04)	0.646	

F/A, number of people with fracture (F) vs. number of all people (A); BMI, body mass index; WC, waist circumference. Model was adjusted for age, gender, race/ethnicity, educational level, poverty income ratio (PIR), drinking behavior, vitamin D, serum calcium, serum phosphorus, total protein, serum uric acid, cholesterol and blood urea nitrogen. The bold values indicate *p* < 0.05, signifying statistical significance.

**Figure 2:**
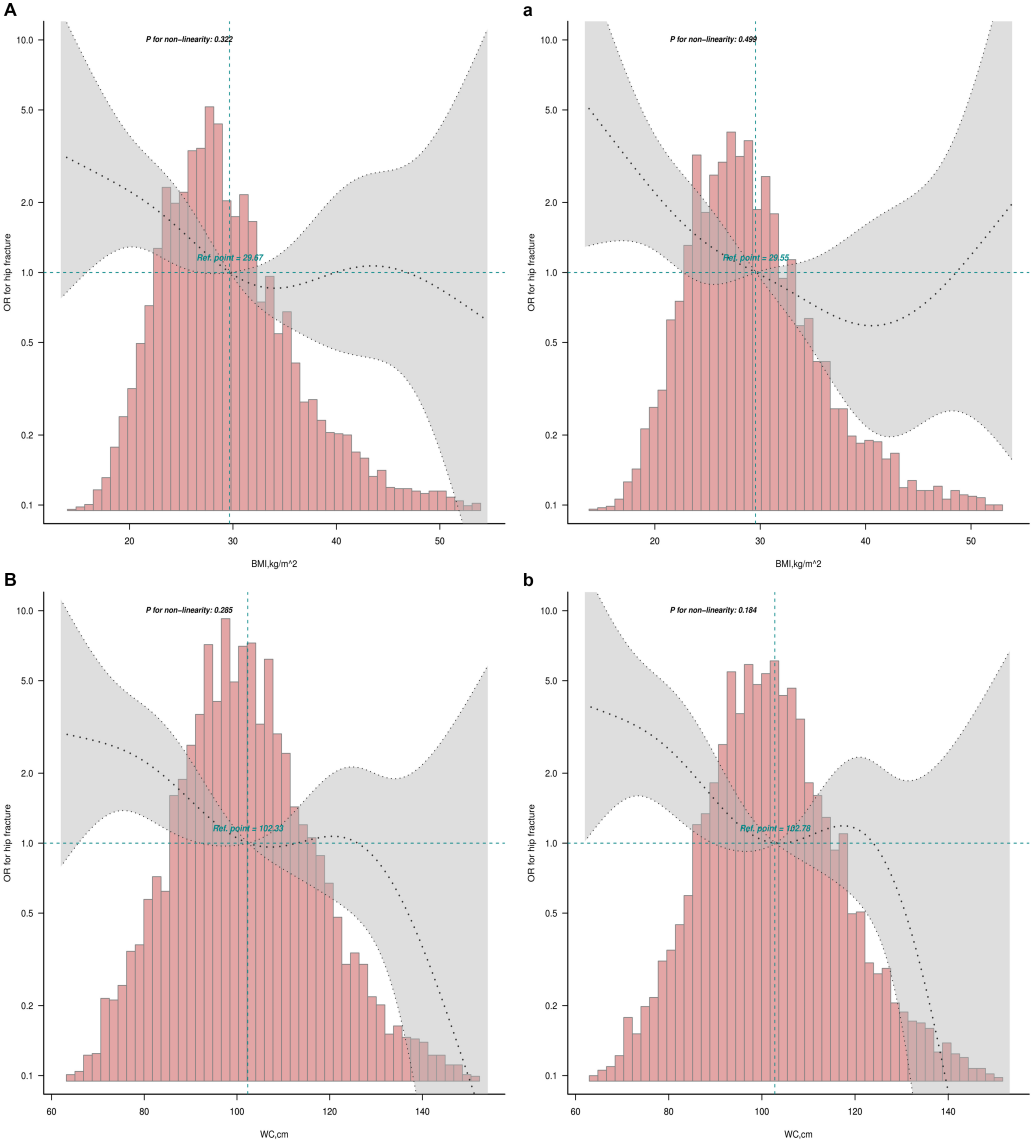
Linear dose–response analysis on BMI and WC and hip fracture. Restricted cubic spline regression with 5 knots at the 5, 27.5, 50, 72.5 and 95th percentiles was used to explore the potential dose–response relationship between BMI and WC and hip fracture. **(A)** The relationship between BMI and hip fracture in all participants; **(a)** the relationship between BMI and hip fracture in participants ≥50 years; **(B)** the relationship between WC and hip fracture in all participants; **(b)** the relationship between WC and hip fracture in participants ≥50 years.

**Figure 3:**
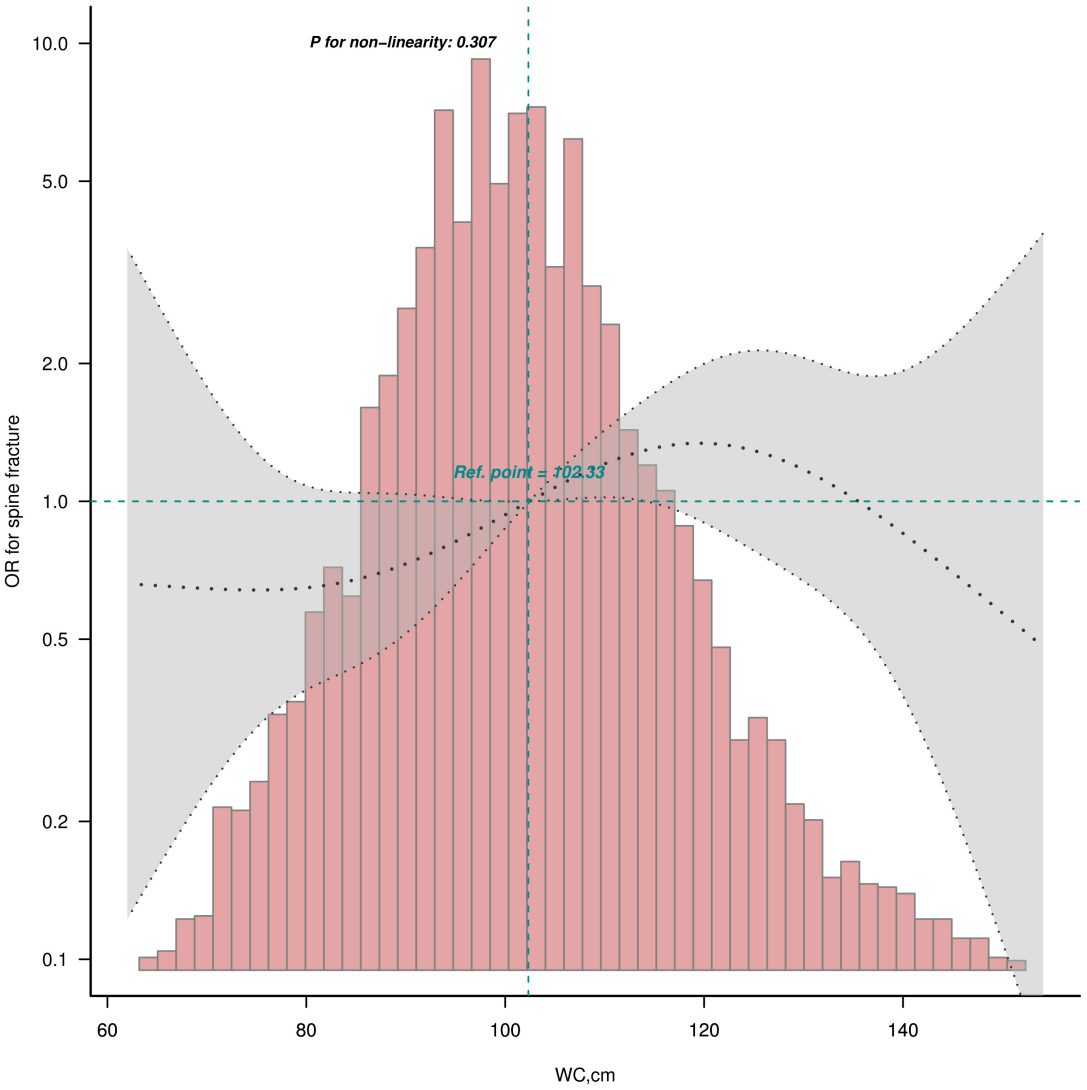
Linear dose–response analysis on WC and spine fracture in all participants. Restricted cubic spline regression with 5 knots at the 5, 27.5, 50, 72.5 and 95th percentiles was used to explore the potential dose–response relationship between WC and spine fracture in all participants.

The smooth curves fitting of the relationship between obesity indices (BMI and WC) and osteoporotic fractures (hip and spine) are shown in Figure 2. The fully adjusted smooth plot showed that a higher BMI and WC was associated with lower risk of hip fracture in all participants, including those aged ≥50 years (all *p* < 0.05) in a linear dose–response manner (all P for non-linearity >0.05; Figure 2). However, a higher WC was observed to be associated with a higher risk of spine fracture in all participants (*p* = 0.019) in a linear dose–response manner (P for non-linearity = 0.307; Figure 3).

WC was analyzed to have a harmful association with spine fracture (OR = 1.43 95%CI: 1.03 ~ 1.99, *p* = 0.034). However, WC defining obesity was found to have no correlation with spine fracture (OR = 1.45 95%CI: 0.99 ~ 2.12, *p* = 0.057; Table 2). Then, WC was further divided into quartiles for males and females separately. Participants in the highest WC quartile were found to have higher risk of spine fracture in unadjusted and adjusted models (OR = 1.53 95%CI: 1.04 ~ 2.26, *p* = 0.031, P for trend = 0.027; OR = 1.99 95%CI: 1.22 ~ 3.24, *p* = 0.006, P for trend = 0.006; Table 4). No interaction was found for age, sex, race, and alcoholic drinks in the correlation between the highest WC quartile and spine fracture (all P for interaction ≥0.05; Supplementary Table S2).

**Table 4:** The association between WC quartiles and spine fracture.

	F/A (%)	OR (95%CI) Model I	p*	OR (95%CI) Model II	p**
WC-1	44/1337 (3.3)	1 (Ref)		1 (Ref)	
WC-2	48/1347 (3.6)	1.09 (0.72 ~ 1.65)	0.698	1.4 (0.86 ~ 2.3)	0.177
WC-3	51/1342 (3.8)	1.16 (0.77 ~ 1.75)	0.476	1.58 (0.96 ~ 2.58)	0.069
WC-4	67/1351 (5)	1.53 (1.04 ~ 2.26)	**0.031**	1.99 (1.22 ~ 3.24)	**0.006**
Trend. test	210/5377 (3.9)	1.15 (1.02 ~ 1.3)	**0.027**	1.24 (1.06 ~ 1.44)	**0.006**

F/A, number of people with fracture (F) vs. number of all people (A). WC, waist circumference; WC-1: < 95.0 cm in male, < 89.2 cm in female; WC-2: 95.0 ~ 104.8 cm in male, 89.2 ~ 99.2 cm in female; WC-3: 104.8 ~ 115.3 cm in male, 99.2 ~ 110.0 in female; WC-4: ≥ 115.3 cm in male, ≥ 110.0 cm in female. Model I: Unadjusted model (univariate model). Model II: Adjusted for age, gender, race/ethnicity, educational level, poverty income ratio (PIR), drinking behavior, vitamin D, serum calcium, serum phosphorus, total protein, serum uric acid, cholesterol and blood urea nitrogen. *p-values were calculated using univariate logistic regression analysis. **p-values were calculated using multivariable logistic regression analysis. *p*-values for trend were calculated using the Cochran-Armitage Trend Test. The bold values indicate *p* < 0.05, signifying statistical significance.

### 3.3 Subgroup analyses on the association of obesity diagnosed by BMI and WC with Hip and spine fractures

Age-specific stratified analyses revealed a protective association for obesity diagnosed by BMI and WC and hip fracture within participants ≥50 years (OR = 0.6 95%CI: 0.37 ~ 0.97, *p* = 0.036; OR = 0.41 95%CI: 0.26 ~ 0.63, *p* < 0.001). No significant interaction between age (Ref: age < 50 years) and obesity diagnosed by BMI and WC for hip fracture was detected (P for interaction = 0.337; P for interaction = 0.055). Sex-specific stratified analyses revealed a protective association for obesity diagnosed by BMI and WC and hip fracture within female (OR = 0.55 95%CI: 0.32 ~ 0.95, *p* = 0.031; OR = 0.34 95%CI: 0.21 ~ 0.57, *p* < 0.001). No significant interaction between sex (Ref: female) and obesity diagnosed by BMI and WC for hip fracture was detected (P for interaction = 0.762; P for interaction = 0.095). Race-specific stratified analyses revealed a protective association for obesity diagnosed by BMI and WC and hip fracture within Non-Hispanic White (OR = 0.46 95%CI: 0.27 ~ 0.8, *p* = 0.005; OR = 0.36 95%CI: 0.22 ~ 0.95, *p* < 0.001). No significant interaction between race (Ref: Mexican American) and obesity diagnosed by BMI and WC for hip fracture was detected (P for interaction = 0.068; P for interaction = 0.485). Drinking behavior-specific stratified analyses revealed a protective association for obesity diagnosed by BMI and WC and hip fracture within no 4/5 or more drinks every day (OR = 0.45 95%CI: 0.25 ~ 0.81, *p* = 0.008; OR = 0.4 95%CI: 0.24 ~ 0.68, *p* = 0.001). No significant interaction between drinking behavior (Ref: 4/5 or more drinks every day) and obesity diagnosed by BMI and WC for hip fracture was detected (P for interaction = 0.198; P for interaction = 0.373; Supplementary Table S1).

Age-specific stratified analyses revealed a dangerous association for obesity diagnosed by BMI and spine fracture within participants ≥50 years (OR = 1.44 95%CI: 1 ~ 2.07, *p* = 0.036). No significant interaction between age (Ref: age < 50 years) and obesity diagnosed by BMI for spine fracture was detected (P for interaction = 0.647). There was insufficient evidence to suggest age, sex, race and drinking behavior could influence the association between obesity diagnosed by BMI and spine fracture. Drinking behavior-specific stratified analyses revealed a dangerous association for obesity diagnosed by WC and spine fracture within participants not consuming 4/5 or more drinks every day (OR = 1.65 95%CI: 1.02 ~ 2.69, *p* = 0.043). No significant interaction between drinking behavior (Ref: 4/5 or more drinks every day) and obesity diagnosed by WC for spine fracture was detected (P for interaction = 0.332). There was insufficient evidence to suggest age, sex, race and drinking behavior could influent the association between obesity diagnosed by WC and spine fracture (Supplementary Table S1).

Obesity diagnosed by BMI and WC was associated with lower odds of hip fracture (OR = 0.31 95%CI: 0.11 ~ 0.88, *p* = 0.027; OR = 0.29 95%CI: 0.11 ~ 0.72, *p* = 0.008) among participants ≥50 years compared with <50 years. Obesity diagnosed by BMI and WC was associated with lower odds of hip fracture (OR = 0.3 95%CI: 0.14 ~ 0.68, *p* = 0.003; OR = 0.4 95%CI: 0.21 ~ 0.76, *p* = 0.005) among participants who do not consume 4/5 or more drinks every day compared with those who do (Supplementary Tables S3, S4).

## 4 Discussion

In this large cross-sectional study for people with diseases influencing bone metabolism, we found that higher BMI and WC were associated with lower risk of hip fracture. Bouxsein et al. observed that the correlation between BMI and trochanteric thickness was notably stronger than the correlation between BMI and total femoral bone density. In other words, individuals with a higher BMI exhibited a close association with thicker trochanteric soft tissue, thereby reducing the risk of hip fractures (26). We inferred that the protective connection between increased WC and hip fracture may stem from the frequent co-occurrence of elevated WC and a high BMI. It is essential to consider the potential role of confounders in our findings. Variables such as physical activity, dietary habits, smoking, and medication use, which were not adjusted for in this study, could influence the relationship between obesity and fracture risk. For instance, individuals with higher physical activity levels generally have better bone health and may exhibit a lower risk of fractures, regardless of their BMI or WC. Similarly, dietary habits, such as calcium and vitamin D intake, can significantly impact bone density and fracture risk. These unmeasured lifestyle factors may have contributed to the observed associations. Additionally, subgroup analyses further showed that the protective relation between BMI and WC and hip fracture was more pronounced in people ≥50 years than <50 years. This finding suggested that the impact of BMI and WC on reducing the risk of hip fractures was particularly significant in the older population. It could be attributed to age-related factors, such as changes in bone density and muscle mass. These results underscored the importance of considering age as a crucial factor when assessing the relationship between body composition and hip fracture risk.

We studied the relative effects of BMI and WC on spine fracture. Our study revealed that a high BMI did not have a significant association with spine fracture. However, a significant correlation was observed between obesity diagnosed by BMI and an increased risk of spine fracture. Conversely, an elevated WC was significantly linked to an increased risk of spine fracture, even though obesity diagnosed by WC showed no relationship with the risk of spine fracture. Meanwhile, we found that the increase in WC level significantly increased the risk of spine fracture in a linear dose–response manner. Further investigation, with WC stratified into quartiles for males and females, revealed a significant correlation between the highest WC quartile and an increased risk of spine fracture. In populations with diseases affecting bone metabolism, it is suggested that when assessing the risk of spine fracture, the standard obesity criteria based on WC from the general population may no longer apply and should be adjusted accordingly (24). One previous study conducted among elderly women found that high WC was significantly linked to an increased risk of vertebral deformities (27), and vertebral deformities may also be a risk factor for spine fracture.

The effect of BMI and WC diagnosed obesity on spine fracture showed different results. The risk of spine fracture was significantly increased in people with obesity diagnosed by BMI, which was consistent with some previous studies (28–30). However, Jin et al.’s study (17) showed that the risk of spine fracture was significantly increased in people with obesity diagnosed by WC rather than BMI. They further found significant interaction between sex and WC diagnosed obesity for spine fracture, and WC diagnosed obesity appeared to influence the risk of spine fracture only in males. However, we observed that there was no significant correlation between obesity diagnosed by the WC obesity standard in the general population and spine fracture. Further investigation revealed that the highest WC quartile was significantly associated with an increased risk of spine fracture. These results shed light on the complex interplay between obesity, abdominal adiposity, and bone health, suggesting that WC may serve as a valuable indicator for assessing the risk of spine fracture in people with diseases influencing bone metabolism, offering opportunities for more targeted preventive measures and interventions.

BMI and WC were common surrogate measures of adiposity in clinical and public health practice (19). BMI was widely used to assess an individual’s overall body fatness, while WC effectively evaluated abdominal obesity, providing insights into the distribution of fat to some extent. Both high BMI and high WC can indicate an individual’s obesity status, but it is unclear whether BMI and WC, defining obesity, have an additive effect on osteoporotic fractures. We utilized BMI and WC standards within the general population to define obesity and investigated the association between different numbers of obesity indicators and the risk of hip and spine fractures. The results indicated that a higher number of obesity indicators was associated with a reduced risk of hip fracture and an increased risk of spine fracture. Intriguingly, the findings suggested an accumulative effect of obesity, as defined by BMI and WC, on the risk of both hip and spine fractures.

Our study focused on the application of BMI and WC criteria to categorize obesity within the general population. We then delved into the relationship between varying numbers of obesity indicators and the associated risks of hip and spine fractures. The findings were enlightening. It appeared that individuals with a higher number of obesity indicators, encompassing both BMI and WC measurements, demonstrated a significantly reduced risk of hip fracture. This suggested that a greater accumulation of obesity-related factors might provide a protective effect against hip fracture. Conversely, our investigation unveiled a contrasting pattern with vertebral fractures. Individuals with an increased number of obesity indicators were found to be at a higher risk of spine fracture. This observation raised intriguing questions about the multifaceted interplay between obesity and bone health. The findings suggested that while obesity, as defined by both BMI and WC standards, may offer protection against hip fracture, it might concurrently elevate the risk of spine fracture.

Additionally, the role of fat distribution and its impact on falls should be considered. Research has indicated that increased adiposity, particularly abdominal fat, may contribute to balance issues and a higher risk of falls, which are critical factors in fracture risk. Higher levels of abdominal fat are associated with poorer balance and increased fall risk in older adults, subsequently leading to fractures (31). Diet also plays a significant role, especially in aging populations. Malabsorption issues common in older adults can affect nutrient intake and bone health, complicating the relationship between obesity and fracture risk. Proper nutrition, including adequate intake of calcium and vitamin D, is crucial for maintaining bone health and preventing fractures (32). Non-invasive techniques for assessing bone health and fracture risk, particularly in fragile populations, are essential and should be emphasized in future studies, as they provide safer and more accessible means of monitoring bone health in at-risk groups (33). Furthermore, sarcopenic obesity, which combines low muscle mass with high fat mass, is associated with increased fall rates and higher fracture risk in older adults, underscoring the importance of fall prevention (34). A higher number of falls is a predictor of increased likelihood of fractures in older adults, highlighting the need for strategies to prevent falls (35).

There were several strengths in this study. First, this was the first population-based study evaluating the impact of BMI and WC on the risk of osteoporotic fractures at three sites in people with diseases influencing bone metabolism. Second, the large sample size of our study contributes to the stability of the results. Third, our study emphasized the cumulative effect of obesity, as defined by BMI and WC, and its intricate association with bone health. Understanding the nuanced effects of different obesity indicators on specific osteoporotic fracture risks can help refine preventive and intervention strategies. Furthermore, this study highlights the importance of considering multiple aspects of obesity when assessing bone fracture risk in clinical settings.

Limitations of this study should also be noted. Firstly, the causality of the results was insufficient due to the cross-sectional design. While most of the measurements for BMI, WC, and osteoporotic fracture questionnaires were collected simultaneously in this cross-sectional study, we can only gather some indications instead of establishing a direct causal relationship. Secondly, the incidence of osteoporotic fracture was obtained from self-report questionnaires, and there was no information about how the reported osteoporotic fractures were diagnosed. It is possible that not all osteoporotic fractures were reported in the questionnaires, as osteoporotic fracture is generally underdiagnosed. Finally, some studies consider that BMD may fall somewhere in the causal chain between obesity and fractures, but we did not focus on this.

## 5 Conclusion

Our study suggested that higher BMI and WC were associated with a lower risk of hip fracture. However, higher WC was associated with a higher spine fracture. Obesity diagnosed by BMI and WC was associated with a lower risk of hip fracture, while obesity diagnosed by BMI and the highest WC quartile were associated with a higher risk of spine fracture. Age can affect the relationship between BMI, WC, and hip fracture, and the protective association is more significant in people aged ≥50 years. Individuals with two indices of obesity were at a lower risk of hip fracture and a higher risk of spine fracture. The binary logistic regression equation established in this study may be clinically useful for predicting osteoporotic fracture risks in populations with diseases affecting bone metabolism.

## Data availability statement

Publicly available datasets were analyzed in this study. This data can be found at: https://www.cdc.gov/nchs/nhanes/index.htm.

## Ethics statement

The studies involving humans were approved by the National Center for Health Statistics Ethics Review Board. The studies were conducted in accordance with the local legislation and institutional requirements. The participants provided their written informed consent to participate in this study.

## Author contributions

GY: Conceptualization, Data curation, Writing – original draft, Writing – review & editing. HJ: Conceptualization, Data curation, Investigation, Methodology, Visualization, Writing – original draft, Writing – review & editing. DX: Conceptualization, Software, Supervision, Validation, Writing – original draft. SY: Investigation, Methodology, Project administration, Resources, Writing – review & editing. JW: Methodology, Resources, Software, Validation, Writing – review & editing. JZ: Data curation, Formal analysis, Investigation, Supervision, Writing – review & editing. LZ: Formal analysis, Methodology, Project administration, Software, Writing – review & editing. JY: Investigation, Project administration, Software, Supervision, Writing – review & editing. JL: Investigation, Project administration, Software, Supervision, Writing – review & editing. JC: Data curation, Investigation, Project administration, Visualization, Writing – review & editing. YY: Conceptualization, Data curation, Funding acquisition, Methodology, Resources, Writing – review & editing.

## Footnotes

^1^ http://www.R-project.org
