# Supplementary material for: Association of obesity with osteoporotic fracture risk in individuals with bone metabolism-related conditions: a cross sectional analysis
Source: Front Nutr. 2024 Aug 6;11:1365587. doi: 10.3389/fnut.2024.1365587 (PMC11333327; doi:10.3389/fnut.2024.1365587)
Supplement: Supplementary file 1 [file Table_1.docx]

**Table S1** Subgroup analyses on the association between obesity and fractures of the hip and spine

|  | Obesity diagnosed by BMI | | |  | Obesity diagnosed by WC | | |  |
| --- | --- | --- | --- | --- | --- | --- | --- | --- |
|  | F/A (%) | OR (95CI%) | P | P for interaction | F/A (%) | OR (95CI%) | P | P for interaction |
| **hip fracture** | | | | | | | | |
| **Subgroup analysis stratified by age** | | | | | | | | |
| Age < 50 | 7/400 (1.8) | 0.79 (0.23~2.68) | 0.702 | 0.337 | 13/582 (2.2) | 1.39 (0.36~5.29) | 0.632 | 0.055 |
| Age >= 50 | 30/1884 (1.6) | 0.6 (0.37~0.97) | **0.036** |  | 57/3198 (1.8) | 0.41 (0.26~0.63) | **<0.001** |  |
| **Subgroup analysis stratified by gender** | | | | | | | | |
| Male | 13/812 (1.6) | 0.76 (0.36~1.58) | 0.457 | 0.762 | 23/1225 (1.9) | 0.78 (0.4~1.54) | 0.479 | 0.095 |
| Female | 24/1472 (1.6) | 0.55 (0.32~0.95) | **0.031** |  | 47/2555 (1.8) | 0.34 (0.21~0.57) | **<0.001** |  |
| **Subgroup analysis stratified by race** | | | | | | | | |
| Mexican American | 5/288 (1.7) | 2.13 (0.46~9.8) | 0.333 | 0.068 | 6/436 (1.4) | 0.6 (0.11~3.33) | 0.559 | 0.485 |
| Other Hispanic | 5/149 (3.4) | 6.97 (0.84~57.72) | 0.072 |  | 5/251 (2) | 1.01 (0.13~7.97) | 0.993 |  |
| Non-Hispanic White | 21/1282 (1.6) | 0.46 (0.27~0.8) | **0.005** |  | 44/2255 (2) | 0.36 (0.22~0.59) | **<0.001** |  |
| Non-Hispanic Black | 5/473 (1.1) | 1.27 (0.27~5.89) | 0.759 |  | 9/667 (1.3) | 0.86 (0.15~5.01) | 0.865 |  |
| Other Race - Including Multiracial | 1/92 (1.1) | 0 (0~Inf) | 0.996 |  | 6/171 (3.5) | 1.05 (0.11~10.54) | 0.965 |  |
| **Subgroup analysis stratified by 4/5 or more drinks every day (%)** | | | | | | | | |
| Yes | 12/374 (3.2) | 0.92 (0.36~2.32) | 0.86 | 0.198 | 18/547 (3.3) | 0.63 (0.24~1.66) | 0.352 | 0.373 |
| No | 18/1519 (1.2) | 0.45 (0.25~0.81) | **0.008** |  | 40/2545 (1.6) | 0.4 (0.24~0.68) | **0.001** |  |
| Not reported | 7/391 (1.8) | 0.94 (0.32~2.72) | 0.906 |  | 12/688 (1.7) | 0.45 (0.16~1.3) | 0.14 |  |
| **Spine fracture** | | | | | | | | |
| **Subgroup analysis stratified by age** | | | | | | | | |
| Age < 50 | 15/400 (3.8) | 1.25 (0.48~3.24) | 0.646 | 0.647 | 24/582 (4.1) | 2.53 (0.88~7.29) | 0.086 | 0.314 |
| Age >= 50 | 89/1884 (4.7) | 1.44 (1~2.07) | **0.048** |  | 129/3198 (4) | 1.32 (0.87~2) | 0.186 |  |
| **Subgroup analysis stratified by gender** | | | | | | | | |
| Male | 43/812 (5.3) | 1.52 (0.93~2.48) | 0.098 | 0.902 | 57/1225 (4.7) | 1.5 (0.89~2.52) | 0.125 | 0.351 |
| Female | 61/1472 (4.1) | 1.36 (0.86~2.14) | 0.187 |  | 96/2555 (3.8) | 1.52 (0.83~2.78) | 0.176 |  |
| **Subgroup analysis stratified by race** | | | | | | | | |
| Mexican American | 10/288 (3.5) | 2.92 (0.81~10.56) | 0.102 | 0.666 | 11/436 (2.5) | 0.95 (0.22~4.03) | 0.946 | 0.405 |
| Other Hispanic | 3/149 (2) | 0.67 (0.1~4.35) | 0.678 |  | 5/251 (2) | 0.29 (0.05~1.77) | 0.181 |  |
| Non-Hispanic White | 69/1282 (5.4) | 1.32 (0.89~1.97) | 0.165 |  | 108/2255 (4.8) | 1.57 (0.98~2.51) | 0.06 |  |
| Non-Hispanic Black | 13/473 (2.7) | 2.01 (0.59~6.83) | 0.263 |  | 15/667 (2.2) | 0.84 (0.21~3.39) | 0.812 |  |
| Other Race - Including Multiracial | 9/92 (9.8) | 2.36 (0.58~9.59) | 0.23 |  | 14/171 (8.2) | 5.91 (1.15~30.28) | **0.033** |  |
| **Subgroup analysis stratified by 4/5 or more drinks every day (%)** | | | | | | | | |
| Yes | 27/374 (7.2) | 1.7 (0.82~3.51) | 0.152 | 0.873 | 35/547 (6.4) | 1.5 (0.66~3.44) | 0.336 | 0.332 |
| No | 66/1519 (4.3) | 1.31 (0.87~1.96) | 0.194 |  | 104/2545 (4.1) | 1.65 (1.02~2.69) | **0.043** |  |
| Not reported | 11/391 (2.8) | 1.87 (0.65~5.35) | 0.246 |  | 14/688 (2) | 0.49 (0.16~1.52) | 0.218 |  |

F/A: number of people with fracture (F) vs. number of all people (A); BMI: body mass index; WC: waist circumference.

Model was adjusted for age, gender, race/ethnicity, educational level, poverty income ratio (PIR), drinking behavior, vitamin D, serum calcium, serum phosphorus, total protein, serum uric acid, cholesterol and blood urea nitrogen.

**Table S2** Subgroup analyses on the association between the highest WC quartile and spine fracture

|  | F/A (%) | OR (95CI%) | P | P for interaction |
| --- | --- | --- | --- | --- |
| **Subgroup analysis stratified by age** | | | | |
| Age < 50 | 6/216 (2.8) | 0.62 (0.2~1.87) | 0.394 | 0.226 |
| Age >= 50 | 61/1135 (5.4) | 1.58 (1.08~2.31) | **0.018** |  |
| **Subgroup analysis stratified by gender** | | | | |
| Male | 30/530 (5.7) | 1.75 (1.04~2.94) | **0.036** | 0.927 |
| Female | 37/821 (4.5) | 1.34 (0.82~2.17) | 0.238 |  |
| **Subgroup analysis stratified by race** | | | | |
| Mexican American | 4/142 (2.8) | 1.26 (0.34~4.65) | 0.729 | 0.901 |
| Other Hispanic | 3/71 (4.2) | 2.18 (0.27~17.44) | 0.462 |  |
| Non-Hispanic White | 45/795 (5.7) | 1.38 (0.91~2.11) | 0.132 |  |
| Non-Hispanic Black | 9/291 (3.1) | 2.09 (0.66~6.61) | 0.209 |  |
| Other Race - Including Multiracial | 6/52 (11.5) | 1.78 (0.36~8.72) | 0.477 |  |
| **Subgroup analysis stratified by 4/5 or more drinks every day (%)** | | | | |
| Yes | 17/244 (7) | 1.57 (0.75~3.29) | 0.231 | 0.894 |
| No | 43/882 (4.9) | 1.36 (0.88~2.11) | 0.165 |  |
| Not reported | 7/225 (3.1) | 1.71 (0.57~5.08) | 0.338 |  |

F/A: number of people with fracture (F) vs. number of all people (A); WC: waist circumference.

Model was adjusted for age, gender, race/ethnicity, educational level, poverty income ratio (PIR), drinking behavior, vitamin D, serum calcium, serum phosphorus, total protein, serum uric acid, cholesterol and blood urea nitrogen.

**Table S3** Subgroup analyses on the association between BMI defining obesity and fractures of the hip and spine

|  | Not obesity | | | Obesity | | |
| --- | --- | --- | --- | --- | --- | --- |
|  | F/A (%) | OR (95CI%) | P | F/A (%) | OR (95CI%) | P |
| **hip fracture** | | | | | | |
| **Subgroup analysis stratified by age** |  |  |  |  |  |  |
| Age < 50 | 10/492 (2) | 1.01 (0.35~2.86) | 0.991 | 7/400 (1.8) | 1(Ref) |  |
| Age >= 50 | 77/2601 (3) | 0.54 (0.19~1.56) | 0.257 | 30/1884 (1.6) | 0.31 (0.11~0.88) | **0.027** |
| Trend. test | 124/5377 (2.3) | 0.95 (0.91~0.99) | 0.017 |  |  |  |
| **Subgroup analysis stratified by gender** |  |  |  |  |  |  |
| Male | 28/1291 (2.2) | 1.13 (0.61~2.08) | 0.695 | 13/812 (1.6) | 0.74 (0.34~1.61) | 0.449 |
| Female | 59/1802 (3.3) | 1.75 (1.03~2.97) | 0.039 | 24/1472 (1.6) | 1(Ref) |  |
| Trend. test | 124/5377 (2.3) | 0.95 (0.91~0.99) | 0.021 |  |  |  |
| **Subgroup analysis stratified by race** |  |  |  |  |  |  |
| Mexican American | 4/276 (1.4) | 0.77 (0.2~2.94) | 0.707 | 5/288 (1.7) | 1(Ref) |  |
| Other Hispanic | 3/214 (1.4) | 0.58 (0.11~3.08) | 0.526 | 5/149 (3.4) | 1.58 (0.41~6.06) | 0.507 |
| Non-Hispanic White | 65/1921 (3.4) | 1.67 (0.63~4.44) | 0.303 | 21/1282 (1.6) | 0.83 (0.3~2.31) | 0.718 |
| Non-Hispanic Black | 7/443 (1.6) | 0.65 (0.18~2.33) | 0.505 | 5/473 (1.1) | 0.49 (0.13~1.9) | 0.306 |
| Other Race - Including Multiracial | 8/239 (3.3) | 2.35 (0.73~7.52) | 0.151 | 1/92 (1.1) | 0 (0~Inf) | 0.976 |
| Trend. test | 124/5377 (2.3) | 0.95 (0.91~0.99) | 0.021 |  |  |  |
| **Subgroup analysis stratified by 4/5 or more drinks every day (%)** | | |  |  |  |  |
| Yes | 14/447 (3.1) | 0.93 (0.4~2.18) | 0.869 | 12/374 (3.2) | 1(Ref) |  |
| No | 57/2076 (2.7) | 0.69 (0.34~1.4) | 0.305 | 18/1519 (1.2) | 0.3 (0.14~0.68) | **0.003** |
| Not reported | 16/570 (2.8) | 0.57 (0.24~1.35) | 0.203 | 7/391 (1.8) | 0.43 (0.15~1.23) | 0.115 |
| Trend. test | 124/5377 (2.3) | 0.95 (0.91~0.99) | 0.021 |  |  |  |
| **Spine fracture** |  |  |  |  |  |  |
| **Subgroup analysis stratified by gender** |  |  |  |  |  |  |
| Age < 50 | 16/492 (3.3) | 0.84 (0.38~1.85) | 0.664 | 15/400 (3.8) | 1(Ref) |  |
| Age >= 50 | 90/2601 (3.5) | 1.09 (0.49~2.4) | 0.836 | 89/1884 (4.7) | 1.58 (0.74~3.35) | 0.234 |
| Trend. test | 210/5377 (3.9) | 1.04 (1~1.07) | 0.03 |  |  |  |
| **Subgroup analysis stratified by gender** |  |  |  |  |  |  |
| Male | 50/1291 (3.9) | 0.87 (0.55~1.37) | 0.543 | 43/812 (5.3) | 1.21 (0.74~1.98) | 0.441 |
| Female | 56/1802 (3.1) | 0.69 (0.44~1.07) | 0.096 | 61/1472 (4.1) | 1(Ref) |  |
| Trend. test | 210/5377 (3.9) | 1.04 (1~1.07) | 0.034 |  |  |  |
| **Subgroup analysis stratified by race** |  |  |  |  |  |  |
| Mexican American | 5/276 (1.8) | 0.41 (0.13~1.33) | 0.136 | 10/288 (3.5) | 1(Ref) |  |
| Other Hispanic | 8/214 (3.7) | 0.68 (0.23~2.06) | 0.5 | 3/149 (2) | 0.39 (0.08~1.83) | 0.233 |
| Non-Hispanic White | 72/1921 (3.7) | 0.83 (0.4~1.73) | 0.625 | 69/1282 (5.4) | 1.14 (0.55~2.34) | 0.721 |
| Non-Hispanic Black | 10/443 (2.3) | 0.3 (0.1~0.92) | 0.034 | 13/473 (2.7) | 0.54 (0.21~1.37) | 0.192 |
| Other Race - Including Multiracial | 11/239 (4.6) | 1.11 (0.43~2.85) | 0.834 | 9/92 (9.8) | 1.73 (0.59~5.06) | 0.315 |
| Trend. test | 210/5377 (3.9) | 1.04 (1~1.07) | 0.034 |  |  |  |
| **Subgroup analysis stratified by 4/5 or more drinks every day (%)** | | |  |  |  |  |
| Yes | 22/447 (4.9) | 0.68 (0.35~1.33) | 0.26 | 27/374 (7.2) | 1(Ref) |  |
| No | 72/2076 (3.5) | 0.52 (0.3~0.89) | 0.017 | 66/1519 (4.3) | 0.7 (0.41~1.21) | 0.203 |
| Not reported | 12/570 (2.1) | 0.29 (0.13~0.67) | 0.004 | 11/391 (2.8) | 0.52 (0.23~1.19) | 0.122 |
| Trend. test | 210/5377 (3.9) | 1.04 (1~1.07) | 0.034 |  |  |  |

F/A: number of people with fracture (F) vs. number of all people (A); BMI: body mass index.

Model was adjusted for age, gender, race/ethnicity, educational level, poverty income ratio (PIR), drinking behavior, vitamin D, serum calcium, serum phosphorus, total protein, serum uric acid, cholesterol and blood urea nitrogen.

**Table S4** Subgroup analyses on the association between WC defining obesity and fractures of the hip and spine

|  | Not obesity | | | Obesity | | |
| --- | --- | --- | --- | --- | --- | --- |
|  | F/A (%) | OR (95CI%) | P | F/A (%) | OR (95CI%) | P |
| **hip fracture** | | | | | | |
| **Subgroup analysis stratified by age** |  |  |  |  |  |  |
| Age < 50 | 4/310 (1.3) | 0.8 (0.25~2.58) | 0.707 | 13/582 (2.2) | 1(Ref) |  |
| Age >= 50 | 50/1287 (3.9) | 0.72 (0.28~1.88) | 0.5 | 57/3198 (1.8) | 0.29 (0.11~0.72) | **0.008** |
| Trend. test | 124/5377 (2.3) | 0.92 (0.88~0.96) | <0.001 |  |  |  |
| **Subgroup analysis stratified by gender** |  |  |  |  |  |  |
| Male | 18/878 (2.1) | 1.15 (0.63~2.09) | 0.648 | 23/1225 (1.9) | 0.8 (0.44~1.47) | 0.473 |
| Female | 36/719 (5) | 2.86 (1.74~4.68) | <0.001 | 47/2555 (1.8) | 1(Ref) |  |
| Trend. test | 124/5377 (2.3) | 0.92 (0.89~0.96) | <0.001 |  |  |  |
| **Subgroup analysis stratified by race** |  |  |  |  |  |  |
| Mexican American | 3/128 (2.3) | 1.84 (0.45~7.6) | 0.397 | 6/436 (1.4) | 1(Ref) |  |
| Other Hispanic | 3/112 (2.7) | 1.71 (0.33~8.79) | 0.519 | 5/251 (2) | 1.2 (0.33~4.35) | 0.778 |
| Non-Hispanic White | 42/948 (4.4) | 3.27 (1.3~8.23) | 0.012 | 44/2255 (2) | 1.2 (0.49~2.98) | 0.688 |
| Non-Hispanic Black | 3/249 (1.2) | 1.05 (0.25~4.43) | 0.944 | 9/667 (1.3) | 0.66 (0.21~2.1) | 0.48 |
| Other Race - Including Multiracial | 3/160 (1.9) | 1.84 (0.44~7.67) | 0.402 | 6/171 (3.5) | 2.41 (0.7~8.24) | 0.161 |
| Trend. test | 124/5377 (2.3) | 0.92 (0.89~0.96) | <0.001 |  |  |  |
| **Subgroup analysis stratified by 4/5 or more drinks every day (%)** | | |  |  |  |  |
| Yes | 8/274 (2.9) | 1.3 (0.53~3.17) | 0.57 | 18/547 (3.3) | 1(Ref) |  |
| No | 35/1050 (3.3) | 1.06 (0.55~2.04) | 0.862 | 40/2545 (1.6) | 0.4 (0.21~0.76) | **0.005** |
| Not reported | 11/273 (4) | 0.93 (0.38~2.26) | 0.877 | 12/688 (1.7) | 0.41 (0.18~0.94) | 0.035 |
| Trend. test | 124/5377 (2.3) | 0.92 (0.89~0.96) | <0.001 |  |  |  |
| **Spine fracture** |  |  |  |  |  |  |
| **Subgroup analysis stratified by age** |  |  |  |  |  |  |
| Age < 50 | 7/310 (2.3) | 0.46 (0.18~1.17) | 0.102 | 24/582 (4.1) | 1(Ref) |  |
| Age >= 50 | 50/1287 (3.9) | 1 (0.48~2.09) | 0.999 | 129/3198 (4) | 1.32 (0.68~2.57) | 0.414 |
| Trend. test | 210/5377 (3.9) | 1.04 (1~1.08) | 0.049 |  |  |  |
| **Subgroup analysis stratified by gender** |  |  |  |  |  |  |
| Male | 36/878 (4.1) | 0.95 (0.59~1.52) | 0.822 | 57/1225 (4.7) | 1.18 (0.77~1.8) | 0.45 |
| Female | 21/719 (2.9) | 0.56 (0.31~1.02) | 0.058 | 96/2555 (3.8) | 1(Ref) |  |
| Trend. test | 210/5377 (3.9) | 1.04 (1~1.08) | 0.057 |  |  |  |
| **Subgroup analysis stratified by race** |  |  |  |  |  |  |
| Mexican American | 4/128 (3.1) | 0.74 (0.2~2.72) | 0.646 | 11/436 (2.5) | 1(Ref) |  |
| Other Hispanic | 6/112 (5.4) | 1.25 (0.38~4.1) | 0.711 | 5/251 (2) | 0.49 (0.13~1.77) | 0.274 |
| Non-Hispanic White | 33/948 (3.5) | 0.93 (0.43~1.97) | 0.841 | 108/2255 (4.8) | 1.45 (0.74~2.85) | 0.275 |
| Non-Hispanic Black | 8/249 (3.2) | 0.55 (0.17~1.81) | 0.325 | 15/667 (2.2) | 0.56 (0.23~1.37) | 0.206 |
| Other Race - Including Multiracial | 6/160 (3.8) | 0.99 (0.3~3.23) | 0.99 | 14/171 (8.2) | 2.36 (0.97~5.71) | 0.058 |
| Trend. test | 210/5377 (3.9) | 1.04 (1~1.08) | 0.057 |  |  |  |
| **Subgroup analysis stratified by 4/5 or more drinks every day (%)** | | |  |  |  |  |
| Yes | 14/274 (5.1) | 0.76 (0.36~1.62) | 0.48 | 35/547 (6.4) | 1(Ref) |  |
| No | 34/1050 (3.2) | 0.46 (0.26~0.82) | 0.008 | 104/2545 (4.1) | 0.78 (0.49~1.23) | 0.284 |
| Not reported | 9/273 (3.3) | 0.52 (0.22~1.24) | 0.141 | 14/688 (2) | 0.39 (0.19~0.82) | **0.013** |
| Trend. test | 210/5377 (3.9) | 1.04 (1~1.08) | 0.057 |  |  |  |

F/A: number of people with fracture (F) vs. number of all people (A); WC: waist circumference.

Model was adjusted for age, gender, race/ethnicity, educational level, poverty income ratio (PIR), drinking behavior, vitamin D, serum calcium, serum phosphorus, total protein, serum uric acid, cholesterol and blood urea nitrogen.
